# The interplay between m6A RNA methylation and noncoding RNA in cancer

**DOI:** 10.1186/s13045-019-0805-7

**Published:** 2019-11-22

**Authors:** Shuai Ma, Chen Chen, Xiang Ji, Jinbo Liu, Quanbo Zhou, Guixian Wang, Weitang Yuan, Quancheng Kan, Zhenqiang Sun

**Affiliations:** 1 Department of Anorectal Surgery, The First Affiliated Hospital of Zhengzhou University, Zhengzhou, 450052 Henan China; 2 Academy of Medical Sciences, Zhengzhou University, Zhengzhou, 450052 Henan China; 3 Department of Pharmacy, The First Affiliated Hospital of Zhengzhou University, Zhengzhou, 450052 Henan China

**Keywords:** M6A modification, Noncoding RNAs, Cancer, Clinical perspectives

## Abstract

N6-methyladenosine (m6A) methylation, one of the most common RNA modifications, has been reported to execute important functions that affect normal life activities and diseases. Most studies have suggested that m6A modification can affect the complexity of cancer progression by regulating biological functions related to cancer. M6A modification of noncoding RNAs regulates the cleavage, transport, stability, and degradation of noncoding RNAs themselves. It also regulates cell proliferation and metastasis, stem cell differentiation, and homeostasis in cancer by affecting the biological function of cells. Interestingly, noncoding RNAs also play significant roles in regulating these m6A modifications. Additionally, it is becoming increasingly clear that m6A and noncoding RNAs potentially contribute to the clinical application of cancer treatment. In this review, we summarize the effect of the interactions between m6A modifications and noncoding RNAs on the biological functions involved in cancer progression. In particular, we discuss the role of m6A and noncoding RNAs as possible potential biomarkers and therapeutic targets in the treatment of cancers.

## Background

The genome-wide distribution of n6-methyladenosine (m6A) was unclear until 2012, but m6A is the most common RNA modification of mRNAs. It is enriched in many eukaryotic species of mammals, plants, and yeast [1–8] and is found in prokaryotes, including bacteria and mycoplasma [9, 10]. The mRNAs of 7676 mammalian genes were found to be modified by m6A [11]. Moreover, there are more than 7000 human genes with 12,000 m6A sites that are enriched in the consensus sequence RRACH (*R* = *G* or *A* and *H* = *A*, *C*, or *U*), which tends to be found in stop codons and 3′ untranslated regions (3′ UTRs) [12]. M6A modifications occur via the m6A methyltransferases called “writers”; they are removed by the demethylases called “erasers” and are recognized by m6A-binding proteins called “readers” [13–16]. M6A modifications are quite prevalent, and the dynamic regulation of m6A modifications has been shown to be significantly related to gene expression [17–22]. Recently, the clinical value of m6A in cancers has become apparent. M6A has been increasingly utilized as a promising biomarker to detect and prevent the occurrence of cancer, and its prognostic significance has been determined [23, 24]. In addition, increasing studies have shown that m6A could have potential clinical applications as therapeutic targets for patients with cancers [25, 26].

Noncoding RNAs consist mainly of microRNAs (miRNAs), long noncoding RNAs (lncRNAs), and circular RNAs (circRNAs) [27, 28]. A common feature of these noncoding RNAs is that they are all transcribed from the genome and can perform biological functions at the RNA level. Importantly, the stable expression of noncoding RNAs in vivo makes them potential biomarkers for the diagnosis, prognosis, and clinical treatment of cancer patients [29, 30].

Recent studies have found that, in addition to the roles of m6A modifications in mRNAs, m6A modifications regulate the generation and function of noncoding RNAs, such as miRNAs, lncRNAs, and circRNAs [31–34]. In addition to controlling their own cleavage, localization, transport, stability, and degradation [13, 14, 35–37], noncoding RNAs regulate the biological functions of cells, including the proliferation, infiltration, and metastasis of certain tumor cells [1, 17–22]. Surprisingly, it was revealed that noncoding RNAs also have a regulatory effect on m6A modifications [38–41]. Thus, concomitant targeting of m6A and noncoding RNAs may provide a synergistic effect in cancer therapy. In this review, we summarize the interactive effects of m6A methylation and noncoding RNAs and describe the way their association influences biological functions in cancer and their possible uses in future clinical applications.

## M6A in cancer

**Fig. 1:**
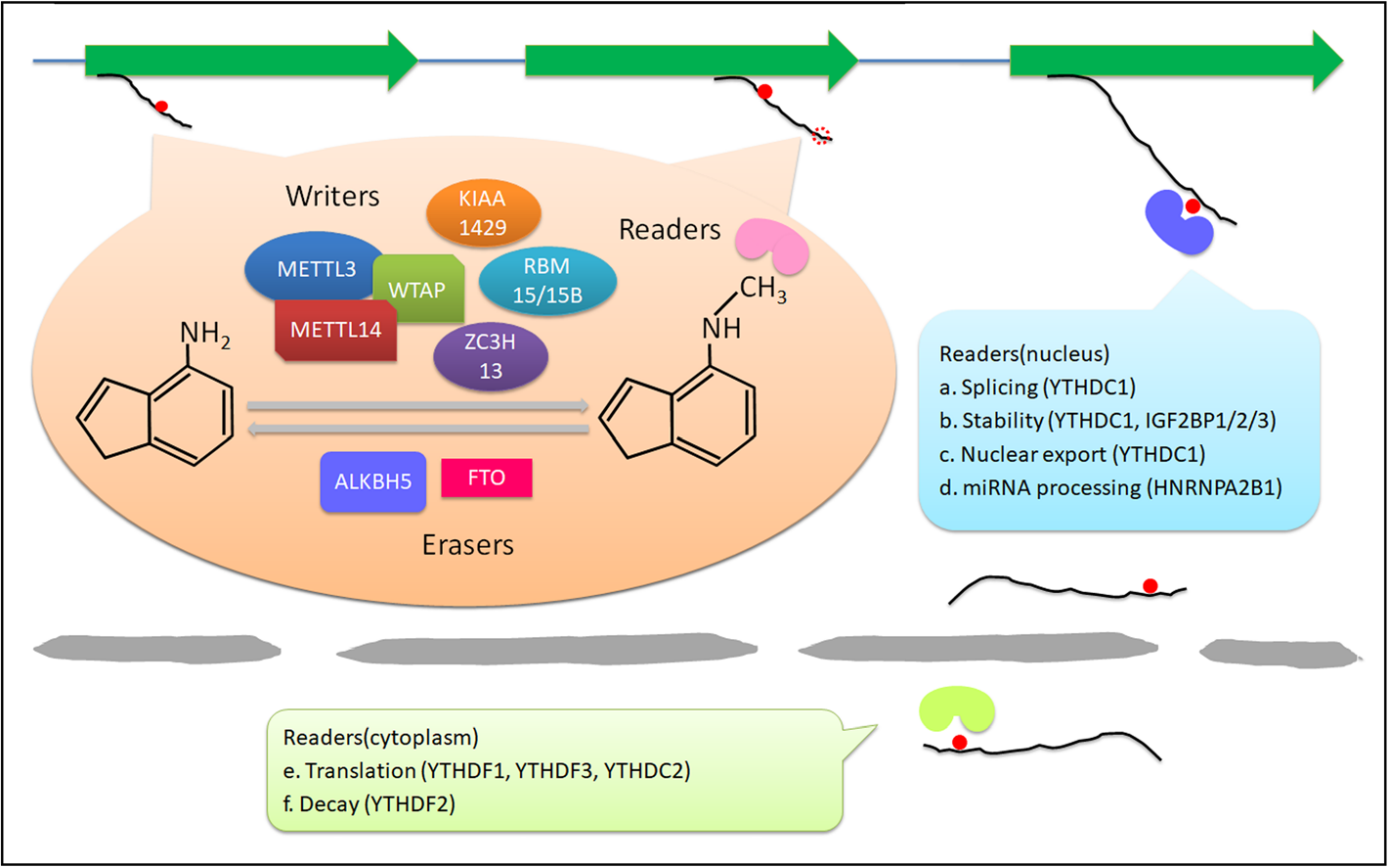
Functions of m6A modifications. M6A modification is a dynamic and reversible process. M6A modifications are catalyzed by the methyltransferase complex consisting of METTL3 and METTL14, as well as their cofactors WTAP, RBM15/15B, KIAA1429, and ZC3H13 (writers). The removal of m6A modifications relies on the demethylases FTO and ALKBH5 (erasers). M6A modifications are functionally facilitated by the m6A binding proteins YTHDF1-3, YTHDC1-2, IGF2BP1-3, and HNRNPA2B1 (readers). **a** YTHDC1 is associated with RNA splicing in the nucleus. **b** YTHDC1 and IGF2BP1-3 are associated with RNA stability in the nucleus. **c** YTHDC1 is associated with RNA nuclear export. **d** HNRNPA2B1 is associated with miRNA processing in the nucleus. **e** YTHDF1, YTHDC2, and YTHDF3 are associated with RNA translation in the cytoplasm. **f** YTHDF2 is associated with RNA decay in the cytoplasm

The m6A modification process is dynamic and reversible in mammalian cells [42]. It has three vital factors: writers, erasers, and readers, which respectively add, remove, or read an m6A site. Methyltransferase-like 3 (METTL3), METTL14, Wilms tumor 1-associated protein (WTAP), KIAA1429, RNA-binding motif protein 15 (RBM15), zinc finger CCCH domain-containing protein 13 (ZC3H13), and METTL16 have been shown to initiate the m6A modification process, which may require readers, such as YTH domain-containing 1 (YTHDC1), YTHDC2, YTH N6-methyladenosine RNA-binding protein 1 (YTHDF1), YTHDF2, and YTHDF3. In contrast, obesity-associated protein (FTO) and alkB homolog 5 (ALKBH5) can stimulate the demethylation process. These regulators have been identified as participants in RNA metabolic processes, including alternative splicing, stability, export, translation, and miRNA processing [13, 14, 35–37] (Fig. 1). These reversible processes are also required for various aspects of viability, embryonic stem cell (ESC) differentiation, and progression in diseases, such as cancers, by regulating the biological functions of cells [1, 17–22]. Recently, an increasing number of studies have explored the control of mRNA metabolism by m6A modifications, showing dual characteristics of m6A modifications and the related regulators in cancers, such as leukemia, lung cancer, pancreatic carcinoma, glioblastoma, and hepatoma [20, 42–45] (Table 1).

**Table 1:** Roles of m6A key members in cancers

Proteins	Cancer	Role	Functional classification	mechanism	References
METTL3	Leukemia	Oncogene	Inhibiting differentiation and increasing cell growth in vitro. Inducting differentiation and apoptosis, and put off leukemia in vivo.	Promoting the translation of c-MYC, BCL2, and PTEN	[46]
	Glioblastoma	Tumor suppressor	Suppressing glioblastoma growth, self-renewal, and tumorigenesis	Regulating oncogenes, such as upregulated ADAM19, EPHA3, and KLF4 and tumor suppressors, such as downregulated CDKN2A, BRCA2, and TP53I11	[43]
	Glioblastoma	Oncogene	Reducing the sensitivity to γ-irradiation and reduced DNA repair in vitro and promoting tumor growth in vivo	Enhancing the SOX2 mRNA stability by recruiting of Human antigen R (HuR) on the m6A sites	[24]
	Lung cancer	Oncogene	Promoting growth, survival, and invasion of human lung cancer cells	Promote the protein translation, such as EGFR, TAZ, MAPKAPK2 (MK2), and DNMT3A	[20]
	Lung cancer	Oncogene	Promoting tumor growth in vivo	Enhancing the translation of BRD4 by interacting with eukaryotic translation initiation factor 3 subunit h (eIF3h).	[47]
	Liver cancer	Oncogene	Promoting HCC cell proliferation and migration	Regulating its target, SOCS2	[48]
	Bladder cancer	Oncogene	Promoting malignant transformation of uroepithelial cells and bladder cancer tumorigenesis in vitro and in vivo	Promoting the stability of CPCP1 translation by YTHDF1 preferentially recognizing m6A residues on CPCP1 3′-UTR	[49]
	Bladder cancer	Oncogene	Promoting cell proliferation, invasion, and survival in vitro and tumorigenicity in vivo	Promoting directly the expression of AF4/FMR2 family member 4 (AFF4), two key regulators of NF-κB pathway (IKBKB and RELA) and MYC	[50]
	Ovarian carcinoma	Oncogene	Promoting cell proliferation, focus formation, motility, invasion in vitro and tumor formation in vivo	Enhancing the translation of AXL to promote the EMT process	[51]
	Endometrial cancer	Tumor suppressor	Inhibiting cell proliferation, anchorage-independent growth, colony formation, migration and invasion in vitro and tumor growth and metastases in vivo	Affecting multiple AKT pathway components to stimulate AKT activation, such as PHLPP2 (a negative regulator of AKT activation)	[52]
	Breast cancer	Oncogene	Promoting proliferation and inhibiting apoptosis in vitro	Promoting the expression of HBXIP through m6A modifications and be inhibited by let-7g which could be arrested by HBXIP	[53]
METTL14	Leukemia	Oncogene	Inhibiting differentiation of AML. Promoting self-renewal of leukemia stem/initiation cells	Regulating mRNA stability and translation of MYB and MYC, be inhibited by SPI1	[54]
	Glioblastoma	Oncogene	Promoting glioblastoma growth, self-renewal, and tumorigenesis	Regulating oncogenes, such as upregulated ADAM19, EPHA3, and KLF4 and tumor suppressors, such as downregulated CDKN2A, BRCA2, and TP53I11	[43]
	Endometrial cancer	Tumor suppressor	Inhibiting cell proliferation, anchorage-independent growth, colony formation, migration and invasion in vitro and tumor growth and metastases in vivo	Affecting multiple AKT pathway components to stimulate AKT activation, such as PHLPP2 (a negative regulator of AKT activation)	[52]
	Hepatoma	Tumor suppressor	Inhibiting the migration and invasiveness in vitro and the tumor growth and metastases in vivo	Regulating the miRNA processing by binding to DGCR8	[55]
	Hepatoma	Oncogene	Promoting HCC cell proliferation and migration	Regulating its target, SOCS2	[56]
FTO	Glioblastoma	Tumor suppressor	Suppressing glioblastoma growth, self-renewal, and tumorigenesis	Regulating oncogenes, such as upregulated ADAM19, EPHA3, and KLF4 and tumor suppressors, such as downregulated CDKN2A, BRCA2, and TP53I11	[43]
	Leukemia	Oncogene	Promoting cell transformation and leukemogenesis, inhibiting cell differentiation in AML	Regulating expression of targets such as ASB2 and RARA by reducing m6A levels in these mRNA transcripts	[45]
	Lung cancer	Oncogene	Promoting the tumor progression of lung cancer	Promoting the stability of MZF1 mRNA transcript	[57]
	Cervical squamous cell carcinoma	Oncogene	Promoting the chemo-radiotherapy resistance in vitro and in vivo	Regulating expression of β-catenin by reducing m6A levels and increasing ERCC1 activity	[25]
ALKBH5	Glioblastoma	Oncogene	Promoting proliferation in vitro and GSCs tumorigenesis in vivo	Promoting expression of FOXM1 nascent transcripts by interacting with FOXM1-AS	[44]
	Breast cancer	Oncogene	Promoting capacity for tumor initiation to increase the number of breast cancer stem cells	Strengthening NANOG mRNA stability by catalyzing m6A demethylation in 3′ UTR of NANOG	[21]
YTHDF1	Melanoma and colon cancer	Oncogene	Promoting tumor growth by regulating tumor immune	Promoting the expression of transcripts encoding lysosomal proteases to degradate tumor antigen	[58]
YTHDF2	Liver cancer	Oncogene	Promoting HCC cell proliferation and migration	Interacting with METTL3 to regulate its target, SOCS2	[48]
IGF2BP1	Ovarian and Liver cancer	Oncogene	Promoting tumor cell growth and cell invasion	Enhancing SRF mRNA stability in an m6A-dependent manner	[59]

## M6A modifications and noncoding RNAs

According to their length, noncoding RNAs can be divided into miRNAs, lncRNAs, circRNAs, snRNAs, rRNAs, tRNAs, etc. Recently, in addition to mRNAs, some noncoding RNAs are reportedly regulated by m6A modifications [31–34, 60]. M6A modifications not only affect miRNA, lncRNA, and circRNA cleavage, transport, stability, and degradation processes but also regulate biological cell functions by affecting noncoding RNA expression [61, 62]. Furthermore, in some cases, these noncoding RNAs can influence the interactions between RNAs and between RNAs and the proteins that regulate their specific biological functions [63–66].

### Regulation of miRNAs by m6A modifications

**Fig. 2:**
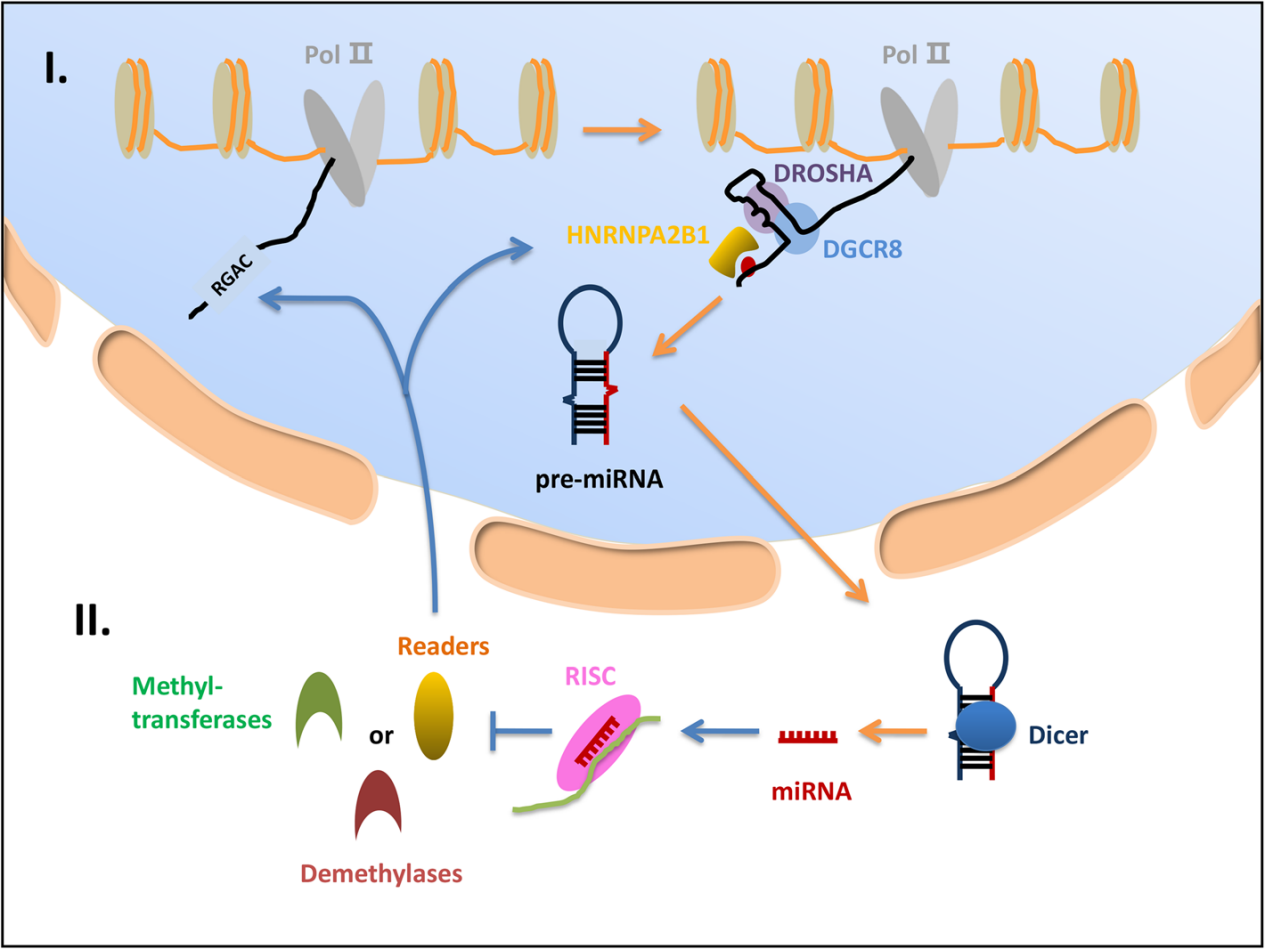
Regulation of miRNAs by m6A modifications. Maturation of miRNA occurs in the nucleus and cytoplasm. **Ι** In the nucleus, the intranuclear enzyme Drosha, an RNase III endonuclease, cleaves primary microRNA (pri-miRNA) to precursor microRNA (pre-miRNA). In addition to Drosha, DGCR8 has also been shown to be critical for miRNA maturation. METTL3-methylated pri-miRNA is recognized and processed by DGCR8. Meanwhile, HNRNPA2B1 recognizes m6A methylation sites. In conclusion, m6A modifications assist DGCR8 in targeting pri-miRNA and promote pre-miRNA formation. **II** Dicer, another RNase III enzyme, cleaves pre-miRNA into mature miRNA after pre-miRNA is transported into the cytoplasm. Then, RISC integrates the mature miRNA and is guided to m6A methylation regulator mRNAs, resulting in disruption of translation by target mRNA cleavage

MiRNAs belong to a class of small noncoding RNAs and are endogenously encoded short RNAs (∼ 21 nucleotides, nt) that are generated from primary transcripts (pri-miRNAs) transcribed by RNA polymerase II/III [67, 68]. MiRNAs are processed by microprocessor and dicing complexes, such as DiGeorge syndrome chromosomal region 8 (DGCR8), which has been reported to play an important role in primary miRNA processing [69, 70]. One study reported that METTL3-methylated pri-miRNAs are recognized and processed by DGCR8. Further experiments suggested that METTL3 can promote miRNA maturation, indicating that these m6A modifications enable DGCR8 to target pri-miRNAs and facilitate miRNA maturation [71] (Fig. 2). METTL3 was also found to indirectly regulate miRNA expression by methylating the hepatitis B virus X-interacting protein (HBXIP). In hepatocellular carcinoma, METTL14 has been identified as a tumor suppressor that coprecipitates with DGCR8. METTL14 depletion leads to miR-126 downregulation and pri-miRNA accumulation. In contrast, METTL14 overexpression induces an increase in the number of pri-miR-126 molecules that are bound to DGCR8 [72–74]. Rescue experiments showed that a miR-126 inhibitor promoted the metastatic ability of METTL14-overexpressing cells, which proved that miR-126 is a key regulator of the process by which METTL14 inhibits hepatocellular carcinoma progression [75].

Another study revealed the role of m6A in miRNA processing, and the role of miR-25-3p was recently reported. In this study, Zang indicated the effect of cigarette smoking on m6A-modified miR-25-3p maturation in pancreatic ductal adenocarcinoma [76]. This research showed that the specific regulatory mechanism of METTL3 upregulation caused by cigarette smoking involves the epigenetic regulation of the METTL3 promoter. This epigenetic modification causes an increase in the METTL3-dependent regulation of pri-miR-25-3p levels and enhances the processing of miR-25-3p. Then, the AKT-p70S6K signaling pathway is influenced after miR-25-3p affects the progression of its target: PH domain leucine-rich repeat protein phosphatase 2 (PHLPP2). Ultimately, the study revealed that the METTL3-miR-25-3p-PHLPP2-AKT regulatory axis might influence the transformation of pancreatic ductal adenocarcinoma induced by cigarette smoking. In addition, the DDX3-dependent network serves as an example of a m6A miRNA modification [77]. DDX3 can lead to m6A RNA demethylation by interacting with ALKBH5. It may also contribute to miRNA demethylation by interacting with the AGO2 protein. In summary, DDX3 function is activated mainly through its interaction with ALKBH5 and AGO2 to regulate cell growth and proliferation. Furthermore, the regulation of miRNAs by m6A modifications also contributes to endocrine resistance in breast cancer cells. HNRNPA2/B1, as a “reader,” can recognize m6A methylation. It was demonstrated that HNRNPA2/B1 expression is higher in breast cancer cells than in normal breast cells, which indicated that HNRNPA2/B1 promotes breast cancer cell proliferation. HNRNPA2/B1 overexpression promotes increases in m6A-modified miRNA levels by enhancing the process by which pri-miRNAs develop into pre-miRNAs and mature miRNAs. Moreover, pre-miRNAs and mature miRNAs can contribute to endocrine resistance by acting on targets or pathways [78]. Knocking down HNRNPA2/B1 can inhibit breast cancer cell proliferation and promote tumor cell apoptosis. Recently, Fish et al. reported that TAR RNA-binding protein 2 (TARBP2), as an RNA-binding protein, recruits a methyltransferase complex to its target transcripts, leading to m6A methylation of transcripts. Then, m6A methylation results in TARBP2-bound transcripts intron retention and nuclear RNA decay. In addition, the authors revealed that TARBP2 promoted lung cancer growth by downregulating the expression of ABCA3 and FOXN3 [79]. RNA-induced silencing complex (RISC) is a 200–500 kDa multiprotein effector complex that has endonuclease activity and integrates mature miRNA. Then, integrated miRNA directs RISC to its target mRNA, causing target mRNA cleavage [80–82]. In addition, TARBP2 is an essential component of the RISC loading complex [83, 84]. Thus, TARBP2-mediated m6A modification may regulate miRNA processes, such as miRNA integration, maturation, and degradation; however, this hypothesis needs to be confirmed by further research.

In summary, miRNA regulation by m6A modifications has an important effect on cancer progression. Thus, we believe that an increasing number of m6A and miRNA regulatory mechanisms will be discovered in the future.

### Regulation of lncRNAs by m6A modifications

LncRNAs are a class of transcripts more than 200 nucleotides long with no protein-coding function [85]. There are 16,193 genes encoding lncRNAs (Gencode v30) in the human genome, and more than 30,000 lncRNA transcripts can be produced. Most lncRNAs have 5′ caps and are spliced and polyadenylated, similar to mRNAs. LncRNAs have m6A modifications and may also regulate gene expression through certain pathways. The effect of m6A modifications on lncRNAs may involve several regulatory mechanisms. On the one hand, m6A modifications might modulate the function of lncRNAs by providing a binding site for the m6A reader proteins or by modulating the structure of the local RNA to induce RNA-binding protein entry. On the other hand, m6A modifications might also regulate the relationship between lncRNAs and specific DNA sites by affecting the RNA-DNA triple helix structure.

**Fig. 3:**
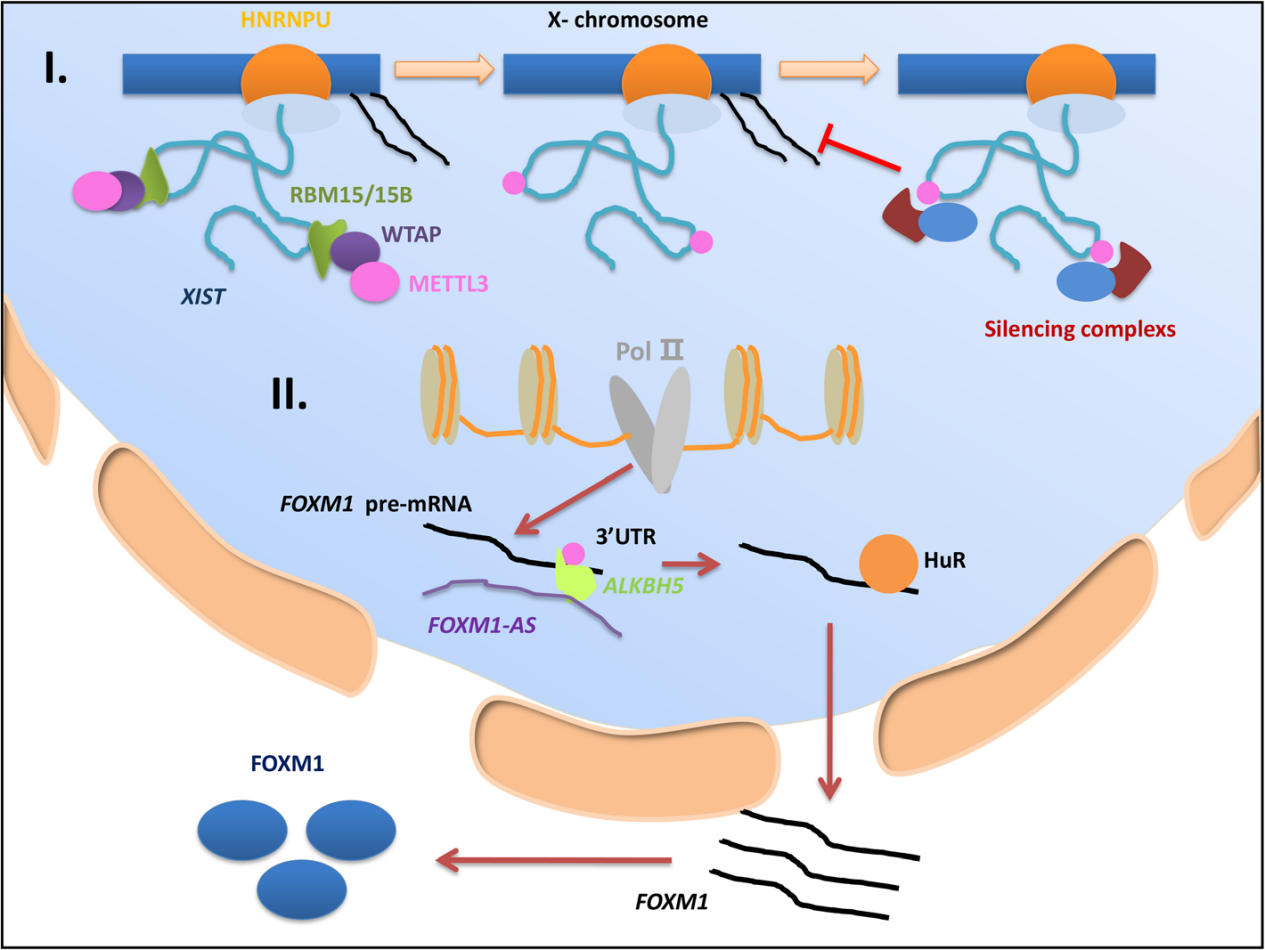
Regulation of lncRNAs by m6A modifications. **Ι**. XIST efficiently silences gene transcription by recruiting specific protein complexes to the X chromosome. XIST regulates the transcriptional silencing of genes by forming the (RBM15)/RBM15B-WTAP-METTL3 complex to recruit the silencing complex. Knocking down METTL3 or RBM15 reduces the level of m6A modifications, leading to impaired XIST-mediated gene silencing. **II** ALKBH5, as an m6A demethylase, interacts with the lncRNA FOXM1-AS to enhance its function. Then, ALKBH5 facilitates the binding of HuR to FOXM1 nascent transcripts. Ultimately, ALKBH5 induces a high level of FOXM1 by demethylating the 3′ UTRs of FOXM1 nascent transcripts

The long noncoding RNA X-inactive-specific transcript (XIST) is proficient at gene-silencing transcription, which it performs by recruiting specific protein complexes to the X chromosome during female mammalian development [86]. A recent study showed that XIST could regulate the transcriptional silencing of genes by forming the RNA-binding protein 15 (RBM15)/RBM15B-WTAP-METTL3 complex, which recruits the silencing complex [64]. In addition, knocking down METTL3 or RBM15 reduced the level of m6A modifications on specific transcripts such that the lncRNA X chromosome was inactivated. Furthermore, YTHDC1 recognized the m6A residues on XIST and prompted subsequent mediation of the regulation initiated by lncRNA-induced gene silencing. In glioblastoma stem cells (GSCs), an m6A demethylase, ALKBH5, was shown to interact with the lncRNA Forkhead box protein M1 (FOXM1)-AS to promote cancer cell proliferation and tumorigenicity. ALKBH5 can induce high levels of FOXM1 transcripts by demethylating FOXM1 nascent transcripts. In this process, the lncRNA FOXM1-AS interacts with ALKBH5, which purportedly enhances the demethylation of the 3′ UTRs of FOXM1 nascent transcripts. However, this research only proved that FOXM1-AS binds with ALKBH5, whether ALKBH5 mediates the demethylation of the FOXM1-AS sequence needs to be further explored [44] (Fig. 3).

Metastasis-associated lung adenocarcinoma transcript 1 (MALAT1) is also a lncRNA that is highly expressed in the nucleus. MALAT1 contains a series of m6A modifications and is upregulated in neoplastic diseases. Studies have confirmed that MALAT1 undergoes structural changes due to m6A modifications, which regulate the interaction between RNAs and some special binding proteins [87]. In addition, m6A modifications of MALAT1 can also affect its localization and activity in the nucleus. M6A modifications of MALAT1 promote the binding of HNRNPG, HNRNPC, or METTL16 to transcripts, which in turn regulates gene expression. LncRNA 1281 is located in the cytoplasm and undergoes frequent m6A modifications. LncRNA 1281 contains several different m6A modification sites, which are essential for the combination of let-7 [33]. It was revealed that altering the m6A modification level of lncRNA 1281 can significantly affect let-7 levels, thereby influencing ESC differentiation. In addition, a recent study revealed that Olfr29-ps1 relies on m6A modifications to exert its biological function. Olfr29-ps1 is a lncRNA pseudogene that is expressed in myeloid-derived suppressor cells (MDSCs) and is regulated by the inflammatory factor interleukin-6 (IL-6). First, GM-CSF and IL-6 induce m6A modifications of Olfr29-ps1. Among these regulators, METTL3 has an important role, as indicated by its specific performance when knocked down; that is, METTL3 downregulation reduces the expression of Olfr29-ps1 in MDSCs, indicating that m6A modifications induce Olfr29-ps1 formation and stability. Then, m6A modifications of Olfr29-ps1 prompt a functional interaction between Olfr29-ps1 and miR-214-3p by recruiting Olfr29-ps1 into the Ago2-related RNA complex. Finally, MyD88 is regulated by miR-214-3p, and knocking down MyD88 exerts an important effect on MDSC immunosuppression and differentiation [88]. In conclusion, Olfr29-ps1 relies mainly on the m6A-modified Olfr29-ps1/miR-214-3p/MyD88 regulatory pathway to modulate MDSC immunosuppression and differentiation [89].

Recently, a number of lncRNAs modified by m6A have been discovered, and they can regulate gene expression and function through a series of complex mechanisms. We believe that other lncRNAs regulated by m6A modifications will be identified in the future.

### Regulation of circRNAs by m6A modifications

CircRNAs were first discovered in an RNA virus in the 1970s, and they belong to the noncoding RNA family lacking 3′ and 5′ ends, which are generally generated by pre-mRNA back-splicing such that they are in the form of loop RNAs [29, 90]. As cyclically structured noncoding RNAs involved in many physiological or pathological processes [91], circRNAs have the characteristics of structural stability, sequence conservation, and tissue-specific expression.

**Fig. 4:**
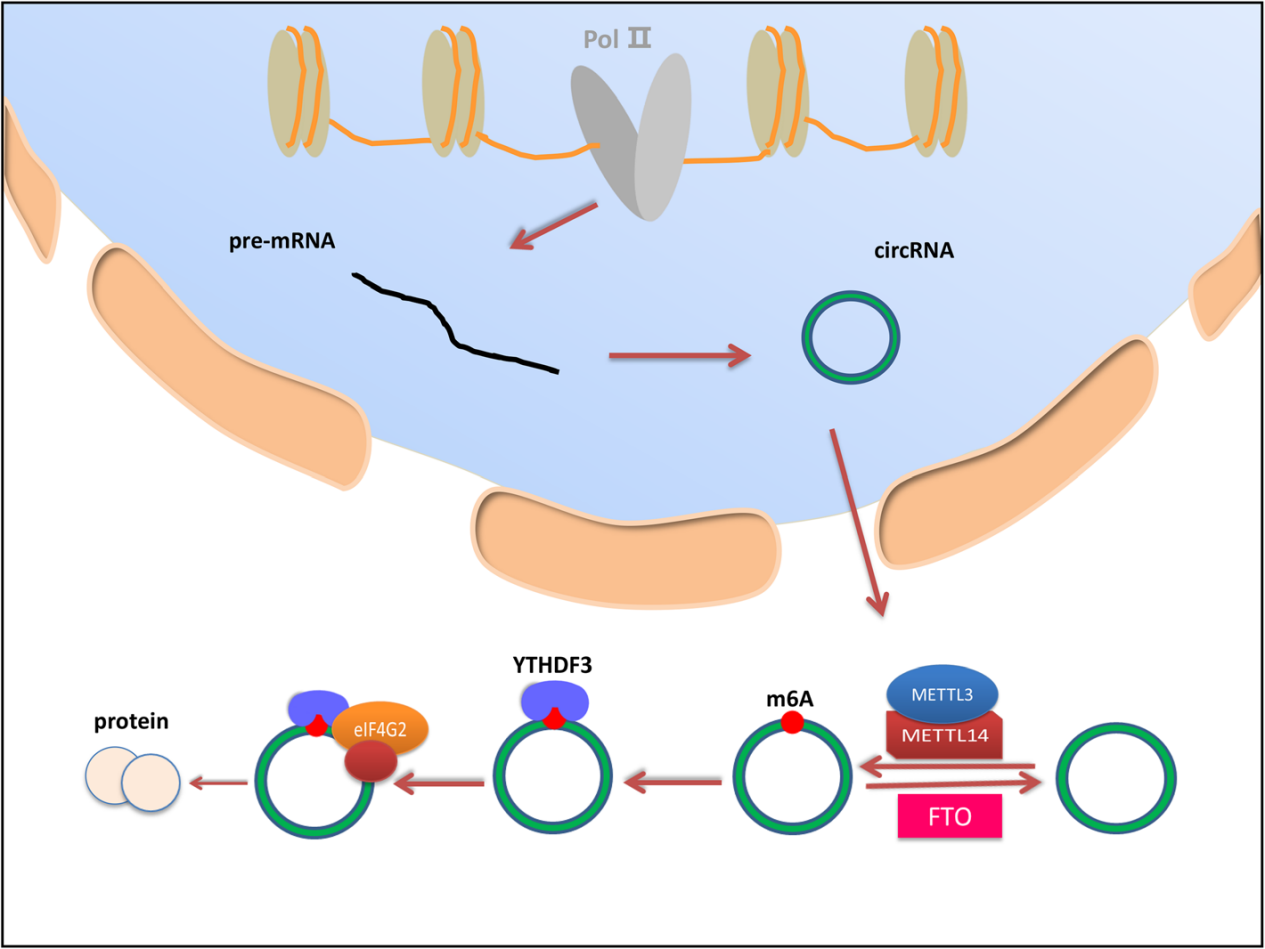
Regulation of circRNAs by m6A modifications. The regulatory mechanism through which m6A modifications affect circRNA occurs in the cytoplasm. CircRNA is regulated by the demethylase FTO and methyltransferase complex METTL3/14. Methyltransferase complex METTL3/14 induces m6A methylation modifications of circRNA, while the demethylase FTO removes m6A methylation of circRNA. YTHDF3 recognizes the m6A methylation site and then recruits eIF4G2 to the circRNA, thus leading to circRNA translation. Therefore, circRNAs can be modified by m6A, and the methylated circRNAs show protein-encoding ability.

Similarly, researchers have found that circRNAs, especially exon-derived circRNAs, can be modified by m6A. Moreover, methylated circRNAs show protein-encoding ability. A study showed that a consistent m6A motif is highly expressed in circRNAs and that a single m6A site can fully drive translation initiation. The study also revealed that circRNAs have m6A modifications, which are regulated by the demethylase FTO and the METTL3/14 methyltransferase complex. Further studies have shown that m6A modifications of circRNAs can promote the translation processes of circRNAs by mediating the eukaryotic translation initiation factor 4G2 (eIF4G2) and binding protein YTHDF3 [31, 65] (Fig. 4). Furthermore, the authors discovered that when circRNAs are in heat shock, their translation function is increased, indicating that circRNA-encoded proteins play an important role in stress environments. In a recent study, Chen et al. demonstrated that m6A modifications of human endogenous circRNAs exerted the important function of inhibiting innate immunity. The authors also revealed that exogenous circRNAs could induce antigen-specific T and B cell activation, antibody production, and antitumor immunity in vivo, while the m6A modifications of these exogenous circRNAs could inhibit immunity activation. In addition, YTHDF2 was essential for inhibiting innate immunity by recognizing m6A [92]. Therefore, these results imply that circRNAs might also regulate tumor progression through their m6A modifications. However, stronger evidence is needed to confirm the regulatory mechanisms involved.

Taken together, these findings suggest that the regulatory mechanisms by which circRNAs interact with m6A members are important to cancer progression and might provide new insights into tumorigenesis and development. However, there are relatively few examples of m6A modifications that regulate circRNAs, and we have not performed a sufficient number of studies to explore these modifications. We strongly believe that the role of m6A modifications in circRNA function will be further revealed by studies on circRNA methylation modifications.

### Regulation of m6A modifications by noncoding RNAs

As explained above, m6A modifications have regulatory effects on noncoding RNAs, including their generation, splicing, transport, degradation, and expression. Interestingly, abnormalities in noncoding RNA levels will also affect m6A levels. For example, miRNAs can influence stem cell differentiation by altering m6A modification levels [38]. In this study, it was revealed that the consensus sequence RRACH (*R* = *G* or *A* and *H* = *A*, *C*, or *U*) motif of m6A sites was reversely complementary to the seed sequences of miRNAs, indicating that, to a certain degree, miRNAs might target m6A peak regions. Notably, miRNAs use a sequence pairing mechanism to regulate m6A modifications. Specifically, miRNAs regulate the binding of METTL3 methyltransferase to mRNAs with miRNA targeting sites to modulate m6A abundance. Then, the increase in m6A abundance induces a series of functions, including the initiation of cell reprogramming that leads to pluripotent mouse embryonic fibroblasts (MEFs). In contrast, reducing the number of m6A modifications can inhibit cell reprogramming. In summary, miRNAs play a significant role in m6A modifications and lay the foundation for cell reprogramming. Another example involves miR-33a, which can affect the proliferation of non-small cell lung cancer (NSCLC) by targeting METTL3 mRNA [39]. METTL3 is a methyltransferase with an important role in m6A modifications. It was found that the METTL3 mRNA expression level was higher in lung cancer tissues than in normal tissues from cancer patients. MiR-33a was shown to be capable of binding directly to the 3′ UTR of METTL3 mRNA in NSCLC cells. However, the miR-33a expression level was negatively correlated with METTL3, and miR-33a could simultaneously cause a decrease in mRNA and METTL3 levels. Then, downregulating METTL3 expression inhibited tumor cell growth and invasion and promoted cell apoptosis [20, 54]. In conclusion, miR-33a exerted tumor-suppressive effects by targeting METTL3 in NSCLC cells. This discovery provides new insights into the mechanisms by which miRNAs regulate m6A modification.

**Fig. 5:**
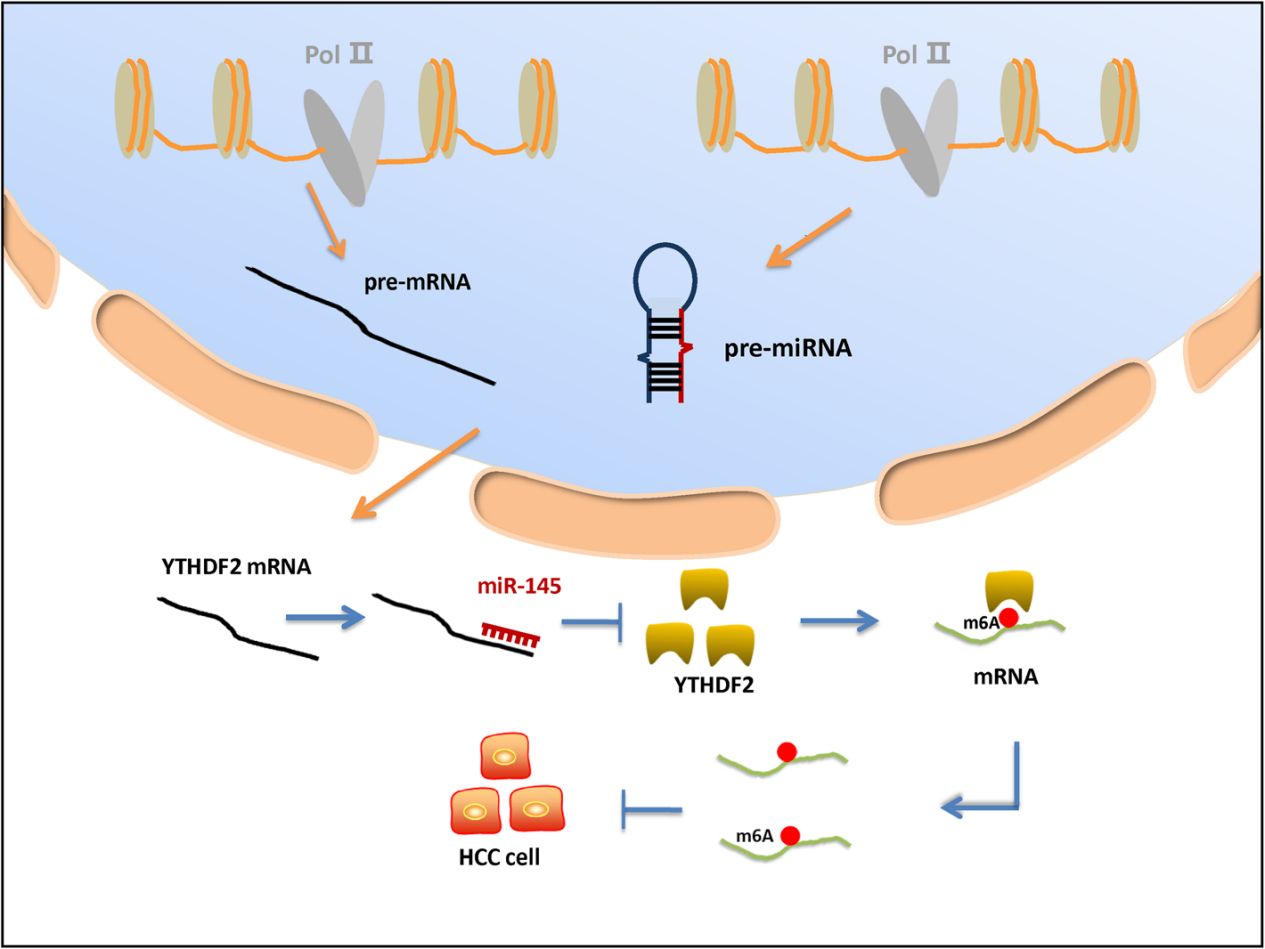
Regulation of m6A modifications by noncoding RNAs. Mature miR-145 and mRNAs are transported to the cytoplasm where they perform their respective roles. MiR-145 reduces the expression of YTHDF2 by targeting the 3′ UTR of YTHDF2 mRNA in HCC cells. Then, the reduction in YTHDF2 increases m6A mRNA levels, leading to decreases in the occurrence, proliferation, invasion, and metastasis of HCC cells. Taken together, the regulation of m6A modifications by miR-145 plays an important role in the biological function of HCC cells

In addition, aberrant expression of mammalian HBXIP, a tumor protein, plays an important role in the occurrence and development of breast cancer [93, 94]. HBXIP is highly expressed in breast cancer and can upregulate METTL3 expression. MiRNA let-7g acted as a tumor suppressor and inhibited tumorigenesis by targeting the 3′ UTR of METTL3 mRNA. HBXIP promoted the expression of METTL3 by inhibiting miRNA let-7g, which resulted in increased m6A modifications. Then, the upregulation of METTL3 expression, in turn, promoted the expression of HBXIP. This regulatory mechanism led to the formation of a positive feedback loop of HBXIP/let-7g/METTL3/HBXIP in breast cancer cells and promoted the occurrence, proliferation, and invasion of breast cancer cells [40]. Furthermore, miR-145 regulated the level and function of m6A modifications by modulating the level of YTHDF2 [34]. Increasing evidence suggests that m6A reader proteins are necessary for m6A modifications to exert their biological functions [95]. YTHDF2 was the first identified m6A reader protein found to regulate mRNA stability. MiR-145 has a variety of biological functions and has been demonstrated to be associated with many human diseases, such as colon, prostate, renal, esophageal, and ovarian cancer [96–100]. It was reported that miR-145 reduced YTHDF2 expression by targeting its 3′ UTR, leading to increased m6A mRNA levels in hepatocellular carcinoma (HCC) cells. Then, YTHDF2 downregulation inhibited the occurrence, proliferation, invasion, and metastasis of HCC cells (Fig. 5). Taken together, the regulation of YTHDF2 by miR-145 plays an important role in the biological function of hepatoma cells. In addition, miR-29a is another example that is worthy of mention. MiR-29a inhibited WTAP and ERK expression by downregulating QKI-6 expression, thereby affecting the PI3K/AKT pathway and inhibiting the occurrence, proliferation, and metastasis of GSCs [41].

In addition to miRNAs, lncRNAs also have a regulatory effect on m6A methylation. LncRNA growth arrest special 5 (GAS5) is a tumor suppressor gene that inhibits the proliferation, invasion, migration, epithelial mesenchymal transition (EMT), and radiation resistance of cancer cells [101–103]. LncRNA GAS5-AS1 is the antisense RNA of GAS5, and its downregulation is closely related to the TNM stage, lymph node metastasis, and prognosis of tumors, such as NSCLC and liver cancer [104, 105]. A recent study reported that GAS5-AS1 enhanced GAS5 stability by acting on the demethylase ALKBH5 and regulating m6A modifications of GAS5, thereby inhibiting the proliferation, invasion, migration, and metastasis of cervical cancer cells. Moreover, m6A-mediated degradation of GAS5 depends on the participation of YTHDF2 [106]. Recently, another study reported that the upregulation of lncRNA ARHGAP5-AS1 promoted chemoresistance in gastric cancer cells. Furthermore, ARHGAP5-AS1 stimulates m6A modifications of ARHGAP5 mRNA by recruiting METTL3 to stabilize ARHGAP5 mRNA in the cytoplasm. Ultimately, ARHGAP5-AS1 enhanced the expression of its target gene ARHGAP5 and promoted chemoresistance in gastric cancer cells. Therefore, targeting the ARHGAP5-AS1/ARHGAP5 axis might serve as a potential therapeutic strategy to overcome chemoresistance in gastric cancer [107].

Studies have confirmed that circRNAs, as miRNA sponges, competitively bind miRNAs and affect their activity and the expression of their downstream target genes [90, 108–110]. MiRNA regulation by circRNAs has been identified in some cancers [111–115]. Moreover, miRNAs have been shown to regulate m6A modifications [40, 41]. Therefore, to some extent, circRNAs might indirectly regulate m6A modifications; however, the mechanisms of these regulatory functions need further confirmation. In summary, with the increasing research on noncoding RNAs and m6A modifications, the regulation of m6A modifications by noncoding RNAs has become apparent, and the associated regulatory mechanisms have been shown to play a significant role in the biological function of cells.

## Potential clinical application of m6A and noncoding RNAs in cancer

### M6A as a potential biomarker and therapeutic target

Most studies have shown that m6A modifications are very common in cancer [24, 75]. For example, Huang et al. have demonstrated that m6A RNA methylation is significantly higher in circulating tumor cells (CTCs) than in whole blood cells, which indicates its role in tumor metastasis. Early detection of m6A upregulation in CTCs plays an important role in monitoring and preventing the occurrence and development of metastatic diseases, indicating that m6A RNA methylation in CTCs is a promising noninvasive diagnostic biomarker for cancer detection [23]. In addition, the Cancer Genome Atlas (TCGA) data analysis revealed that METTL3 was upregulated in multiple cancers, including liver cancer, breast cancer, colorectal cancer, prostate cancer, and bile duct cancer. High METTL3 levels were significantly associated with poor prognosis in patients with HCC [24]. Similar to METTL3, WTAP overexpression is linked to a poor prognosis in malignant glioma patients [116]. The expression of the reader YTHDF1 also indicates a poor prognosis in patients with HCC [117]. In conclusion, as a potential biomarker, m6A could be utilized to predict the diagnosis and prognosis of cancer patients.

In addition, m6A modifications have the potential to be involved in combined tumor therapy. In a previous study, Kwok et al. analyzed TCGA datasets and found that alterations in m6A regulatory genes were closely related to TP53 mutations in acute myeloid leukemia (AML) patients. Furthermore, the analysis results showed that alterations in m6A regulatory genes reduced the survival rate of AML patients. Thus, m6A regulatory genes may be new molecular targets for the treatment of AML [26]. PD-L1 immunotherapy against tumors is well established. Han et al. found that YTHDF1 deletion significantly enhanced the therapeutic efficacy of PD-L1 checkpoint blockade, suggesting that YTHDF1, which plays a key role in m6A modifications, might be a potential therapeutic target in anticancer immunotherapy. Furthermore, m6A regulators play important roles as possible therapeutic interventions for treating cancer. Chemoradiotherapy is the main treatment method for cancer patients after surgical operation or during disease progression. Individual differences lead to different chemoradiation sensitivities. Therefore, it is very important for patients to know their own chemoradiation sensitivity by using an indicator, and an m6A regulator may be such an indicator. Studies have shown that METTL3 loss in pancreatic cancer cells is linked to higher sensitivity to anticancer reagents, such as gemcitabine, 5-fluorouracil, cisplatin, and irradiation. It has been suggested that METTL3 promotes chemoradiation resistance in pancreatic cancer [118]. Additionally, FTO expression levels were found to be higher in CSCC tissue than in adjacent normal tissues. FTO promotes chemoradiotherapy resistance in vitro and in vivo by decreasing m6A modification levels in its targets. Significantly, scholars found that the prognostic value of FTO in CSCC samples was dependent on β-catenin expression, which might indicate that detecting these factors in combination may be important in determining the chemoradiotherapy regimen for a patient [25]. These findings suggest that m6A regulators might be potential molecular targets for patients undergoing chemoradiotherapy. As we discussed, Chuan et al. reported that R-2HG showed antitumor activity by blocking FTO to induce MYC degradation in AML patients with an IDH mutation. TCGA data reveal high MYC levels and low FTO levels in tissue cells with an IDH mutation. The use of R-2HG and MYC inhibitors could enhance cytotoxicity, which indicates that the combined utilization of R-2HG and MYC may be an effective treatment method for patients with leukemia characterized by an IDH mutation [59]. In summary, the analysis of m6A modifications could be used as a potential therapeutic target for cancer treatment.

### Potential clinical application of regulatory mechanisms between m6A and noncoding RNAs

In addition to the clinical application of the m6A modifications described above, intervention through m6A modifications, and/or noncoding RNA regulatory mechanisms at certain carcinogenic sites may affect tumor proliferation. Targeted therapy may also be applied to the regulation of noncoding RNAs.

MiRNAs have been described as promising therapeutic targets in cancers [119, 120]. Poudyal et al. demonstrated that miRNA-6852 overexpression could induce G2/M phase arrest in cervical cancer cells [121]. Zhao et al. revealed that miR-143 might regulate the proliferation and apoptosis of cervical cancer cells by targeting HIF-1α [122]. These results suggest that miRNAs could be potential therapeutic targets for patients with cervical cancer. Increasing studies have also shown that lncRNAs can act as biomarkers for clinical cancer treatment [123]. A study revealed that lncRNA RP11-708H21.4 upregulation not only inhibited the migration and invasion of tumor cells and promoted apoptosis but also enhanced the 5-FU sensitivity of colorectal cancer cells by inactivating the mTOR signaling pathway [124]. Furthermore, lncRNA PVT1 upregulation can promote the proliferation, migration, and invasion of osteosarcoma by regulating the miR-195/FA synthase (FASN) signaling pathway. Silencing lncRNA PVT1 expression can restore the inhibitory effect of miR-195 on FASN and thus inhibit the proliferation, migration, and invasion of osteosarcoma [125]. LncRNA RP11-708H21.4 and lncRNA PVT1 also have potential as therapeutic targets for cancer. The potential clinical application of circRNAs has been gradually highlighted due to biotechnology innovations [126, 127]. A recent study demonstrated that ciRS-7 promoted tumor cell proliferation, migration, and invasion by blocking the miR-7-mediated PTEN/PI3K/AKT signaling pathway [128]. Weng et al. also revealed that ciRS-7 could regulate the EGFR/RAF1/MAPK signaling pathway in colorectal cancer through competition with miR-7 [129]. The above data indicate that ciRS-7 is a promising target for cancer therapy.

Recent studies have discovered that DCGR8 could be recruited by METTL14 to m6A-modified pri-miRNA. After being subjected to a series of regulatory actions, DCGR8 affects the expression of tumor cells in HCC [75]. In this case, as a protein molecule, DGCR8 has potential as a therapeutic target. Another study reported that the upregulation of METTL3 caused an increase in miR-25-3p and that miR-25-3p could promote the expression of its target protein PHLPP2 to promote pancreatic ductal adenocarcinoma occurrence [76]. Similar to DGCR8, the protein PHLPP2 also shows potential for use in targeted therapy. Targeting PHLPP2 specifically could reduce its expression, thereby inhibiting the occurrence and proliferation of tumor cells in pancreatic ductal adenocarcinoma. In addition, it was speculated that noncoding RNAs could be used as breakthrough points in targeted therapy. Targeting the consensus sequence RRACH might block the binding of m6A to noncoding RNAs. The m6A modification of MALAT1 could regulate gene expression. Studies have confirmed that MALAT1 is upregulated in various tumor tissues, such as NSCLC, breast cancer, cervical cancer, and bladder cancer, and is closely associated with the occurrence, development, and metastasis of tumors [87]. A recent study also revealed that altering the modification levels of m6A in lncRNA 1281 could significantly affect ESC differentiation [33]. Therefore, noncoding RNAs have the potential to become new therapeutic targets. By acting specifically on the consensus sequences of noncoding RNAs, the levels of m6A modification transcripts are decreased, which affects the expression of downstream genes, thereby regulating the biological functions of tumor cells. Thus, as a potential target, noncoding RNAs can provide new possibilities for clinical treatment through their associations with m6A modifications. However, the specific mechanism of noncoding RNAs for use in targeted therapy needs to be further confirmed.

## Conclusions

M6A is one of the most common RNA modifications and plays a significant regulatory role in the biological function of cells, especially in cancer. M6A modifications of noncoding RNAs have been demonstrated to control gene expression, as demonstrated by their ability to regulate the biological functions of cells in cancer, including proliferation, metastasis, stem cell differentiation, and homeostasis. Similarly, noncoding RNAs have the ability to regulate m6A modifications, thereby affecting gene expression in cancer progression. The association of m6A modifications and noncoding RNAs provides a new direction for exploring the underlying regulatory mechanisms of gene expression in cancer. Furthermore, the clinical application of m6A modifications and noncoding RNAs includes their use as cancer indicators and targets for therapeutic interventions in cancer treatment. Further studies are needed to explore the mutual regulatory mechanisms between m6A modifications and noncoding RNAs in additional types of cancers and the effective therapeutic interventions of m6A modifications and noncoding RNAs for cancer patients.

## Data Availability

The dataset supporting the conclusions of this article is included within the article.

## Abbreviations

3′ UTRs: 3′ Untranslated regions
AFF4: AF4/FMR2 family member 4
ALKBH5: AlkB homolog 5
AML: Acute myeloid leukemia
BCL2: B cell lymphoma 2
CircRNA: Circular RNA
CTCs: Circulating tumor cells
DGCR8: DiGeorge syndrome chromosomal region 8
EGFR: Epidermal growth factor receptor
eIF3h: Eukaryotic translation initiation factor 3 subunit h
eIF4G2: Eukaryotic translation initiation factor 4G2
EMT: Epithelial mesenchymal transition
FASN: FA synthase
FOXM1: Forkhead box protein M1
FTO: Obesity-associated protein
GAS5: Growth arrest special 5
GSCs: Glioblastoma stem cells
HBXIP: Hepatitis B virus X-interacting protein
HCC: Hepatocellular carcinoma
HuR: Human antigen R
IL-6: Interleukin-6
LncRNA: Long non-coding RNA
m6A: N6-methyladenosine
MALAT1: Metastasis-associated lung adenocarcinoma transcript 1
MDSCs: Myeloid-derived suppressor cells
MEFs: Mouse embryonic fibroblasts
METTL14: Methyltransferase-like 14
METTL16: Methyltransferase-like 16
METTL3: Methyltransferase-like 3
MiRNA: MicroRNA
MZF1: Myeloid zinc finger 1
NSCLC: Non-small cell lung cancer
PHLPP2: PH domain leucine-rich repeat protein phosphatase 2
PTEN: Phosphatase and tension homolog
RBM15: RNA-binding motif protein 15
RISC: RNA-induced silencing complex
SOCS2: Suppressor of cytokine signaling 2
SOX2: Sex determining region Y-box 2
TARBP2: TAR RNA-binding protein 2
TAZ: Tafazzin
TCGA: The Cancer Genome Atlas
WTAP: Wilms tumor 1-associated protein
XIST: X-inactive specific transcript
YTHDC1: YTH domain-containing 1
YTHDF2: YTH N6-methyladenosine RNA-binding protein 2
ZC3H13: Zinc finger CCCH domain-containing protein 13
